# An Association Rule Analysis of the Acupressure Effect on Sleep Quality

**DOI:** 10.1155/2021/1399258

**Published:** 2021-09-29

**Authors:** Chih-Hung Lin, Ya-Hsuan Lin, I-Shiang Tzeng, Chan-Yen Kuo

**Affiliations:** ^1^Respiratory Care and Chest Medicine, Department of Internal Medicine, Cathay General Hospital, Taipei, Taiwan; ^2^School of Medicine, College of Medicine, Fu Jen Catholic University, New Taipei, Taiwan; ^3^Department of Chinese Medicine, Taipei Tzu Chi Hospital, Buddhist Tzu Chi Medical Foundation, New Taipei, Taiwan; ^4^Department of Research, Taipei Tzu Chi Hospital, Buddhist Tzu Chi Medical Foundation, New Taipei, Taiwan; ^5^Department of Statistics, National Taipei University, Taipei, Taiwan; ^6^Department of Nursing, Cardinal Tien College of Healthcare and Management, New Taipei, Taiwan

## Abstract

**Background:**

Sleep is recognized as an all-important physiological process, which also contributes to maintaining several bodily functions and systems. According to the Pittsburgh Sleep Quality Index (PSQI), also known as the most widely used tool in the field of subjective assessment of self-perceived sleep quality, a combination of acupoints could be more effective than single acupoint treatment in improving sleep quality.

**Methods:**

The present study was based on the extracted eligible studies rooted in a previous meta-analysis that worked on the basis of association rule mining and examined the potential kernel acupoint combinations for improving sleep quality.

**Results:**

Depending on the Apriori algorithm, we summarized 26 acupoints as binary data from the 32 eligible studies based on a previous meta-analysis and analyzed them. The top 10 most frequently selected acupoints were HT7, SP6, PC6, KI1, GV20, EM5, EX-HN3, EX-HN16, KI3, and MA-TF1. Furthermore, as deduced from 21 association rules, the primary relevant rules in the combination of acupoints are (EX-HN3, EX-HN16)=>(GV20) and (HT7, KI1)=>(PC6).

**Conclusions:**

In order to use acupuncture to improve sleep quality, integrating (EX-HN3, EX-HN16, GV20) with (HT7, KI1, PC6) acupoints could be deemed as the kernel acupoint combination.

## 1. Introduction

The essentiality of sleep as a vital and intricate physiological process in humans cannot be denied. Over the years, numerous studies [1, 2] have suggested that this process is affected by three elements, which are social, cultural, and environmental in nature. Nowadays, high levels of stress and poor sleep quality [3] result from the social and organizational demands experienced by individuals. Moreover, an increase in the number of diseases related to sleep quality [4] is an outcome of organic disorders. Driving accidents that cause over 2000 fatal crashes and 40,000 nonfatal injuries each year are two instantaneous consequences due to lack of sleep or poor quality of sleep in the US. Belated sequelae caused by sleep disorders are associated with the risk of many diseases such as metabolic disorders [5, 6], psychiatric conditions [7], cardiovascular diseases [8], and even cancers [4]. In addition, poor quality sleep and insomnia have a tight connection with emotions. Previous studies have observed the implications of loneliness, grief, hostility, impulsivity, stress, depression, and anxiety in terms of sleep [1, 9–11]. The close-knit relationship displayed between emotion and sleep is gradually being distinguished as a crucial area for research [12]. In order to understand the processes that can provide good quality sleep [13–16], recent studies have reported some mechanisms of sleep [17, 18] to comprehend its behavioral complexity and advancement beyond pathological descriptions.

In the last few years, the requirements on the estimation of the factors influencing the quality of sleep have increased. The necessity to understand these associations was motivated by the discovery that biological traits do not always have linkages with the perception of poor quality of sleep [19]. The Pittsburgh Sleep Quality Index (PSQI) is a method to investigate the subjective quality of sleep. This implementation provides an accurate picture of seven different circumstances of sleep: (a) sleep duration, (b) sleep disturbance, (c) sleep latency, (d) daytime dysfunction, (e) sleep efficiency, (f) subjective sleep quality, and (g) use of sleep medication [20]. Clinical practice is a better approach here rather than objective sleep measures [21, 22].

Applying pressure to specific points on the body is a traditional treatment known as acupressure. These specific points are referred to as acupoints, which correspond to different organs and systems in the human body. Traditional Chinese medicine (TCM) acupressure is the most empirically studied form of acupressure which is closely connected with TCM acupuncture. Nevertheless, instead of needles, practitioners of TCM acupuncture use fingers, knuckles, or dull. It has received much more attention on the grounds of its safe supplementary and alternative effects that overtly alleviate the symptoms of certain diseases. Therefore, acupressure could be a successful treatment for patients with sleep disorders in the near future. In clinical practice, poor sleep quality is a widely reported complaint. At the same time, it can also be identified as a significant symptom among various sleep and medical disorders [20, 23]. Frankly speaking, sleep quality can be considered as a combination of two conceptions, the quantitative aspects, such as sleep duration and sleep latency, and subjective perceptions of sleep, such as depth and restfulness [20]. In order to evaluate sleep quality [24, 25], subjective and objective assessments are applied as two prevalent, distinct, and complementary approaches in research as well as clinical settings.

As a matter of fact, based on a previous meta-analysis, it is claimed that acupuncture in conjunction with Chinese herbs is a tolerable and effective nonpharmacological treatment [26] for improving sleep quality of patients (i.e., PSQI). This meta-analysis included patients, clinicians, and decision makers with evidence-based advice in the healthcare system. In the meantime, further investigation through proper methodology is encouraged. Recent studies have discussed the relationship between acupuncture points and diseases. Data mining methods, which have been widely utilized in modern fields and Chinese medicine, are being employed to enhance the therapeutic effect of the treatment. A prior study supplied reference based data mining results with the selection and combination of acupuncture points for remedying various sleep disorders by means of clinical acupuncture therapy [27]. Some valuable suggestions about the selection and combination of acupuncture points for sleep disorders have also been contributed by another research [28]. As stated in the literature review, data mining has been used extensively on the scale of discovering potential acupuncture points and treating specific diseases effectively. Using a data mining approach [29], a study examined the selections and characteristics of acupuncture point principles for chronic kidney disease treatment. In reality, association rule mining (analysis) is generally used in the sphere of marketing to determine strong and frequent directional associations between jointly purchased items.

In this study, with the intention of determining the effect of acupuncture on sleep quality, we found the potential kernel combination of acupuncture points in accordance with acupoint data from a previous meta-analysis [26].

## 2. Materials and Methods

### 2.1. Data Sources

Based on a meta-analysis [26], acupoint data integrated acupuncture data that were interpreted by the content of the WHO Standard Acupuncture Point. This study was based on the previously reported meta-analysis study which reviewed 32 eligible studies. From a selection of 32 eligible studies that originated from the abovementioned study, there was an integration of 26 acupuncture point locations. All the included studies were required to use acupuncture-related methods, such as acupuncture, electroacupuncture (EA), abdomen acupuncture, eye acupuncture, ear acupuncture, or scalp acupuncture, and have precise outcome data on quality of sleep. We made a record of 26 acupuncture point locations involved in studies as binary data (Supplementary Table 1).

### 2.2. Risk of Bias Assessment

The Cochrane RoB 2.0 tool was used to assess quality the studies. The Cochrane RoB 2.0 tool investigated risk of selection bias, performance bias, detection bias, attrition bias, and reporting bias. Finally, the tool combined the above bias to assess the quality of 13 randomized controlled trials (RCTs) selected from 32 eligible studies based on a previous meta-analysis [26] (Supplementary Figure 1).

### 2.3. Data Analysis

On the basis of “arules” and “arulesViz” packages, association rule analysis (ARA) and plotting were performed with statistical software *R* (version 4.0.0). Cochrane RoB 2.0 tool was applied to evaluate the methodological quality of the studies included in this meta-analysis [26]. This tool uses seven domains to assess RoB and evaluates the overall quality of RCT after each domain is combined. The association rule learning algorithm is one of the widely used techniques to detect and analyze relations and useful information from transaction data. The association rule learning algorithm contains an antecedent and consequent sets, both of which are a set of items. In this study, support, confidence, expected confidence, and lift were kernel values involved with association rule analysis. First, support means the fraction of the total number of transactions in which the itemset occurs. And confidence defined the conditional probability of occurrence of consequent, given the antecedent. Next, expected confidence presents the probability of the consequent while consequent was independent of the antecedent. Final, lift expressed the ratio of joint antecedent and a consequent probability and product of each marginal probability.

Support and confidence factors are essential parameters in association rule learning. Support estimates the frequency of an acupoint appearing in the 32 formulas. On the other hand, confidence measures the frequency of acupoint appearing in the formulas, given that acupoint B appears simultaneously. Expected confidence is the number of formulas that include the consequent set of acupoints divided by the total number of formulas. During the exploration of the association rules, users need to test multiple combinations of the minimum values for support and confidence factors to discover the significant association rules. However, the selection of thresholds showed slight ambiguity and varied from case to case. If the parameter thresholds were set at extremely high values, then certain meaningful information would be discarded.

## 3. Results

### 3.1. Risk of Bias Assessment

The summary of 13 RCTs selected from 32 eligible studies based on a previous meta-analysis [26] and quality assessment with overall bias is presented in Supplementary Table 2. The results showed no serious risk of bias consisted with the previous study [26].

### 3.2. Acupoint Distribution

**Figure 1:**
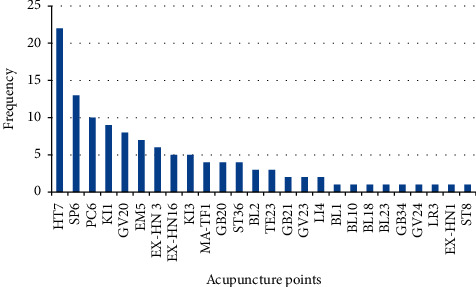
Acupoints distribution extracted from 32 eligible studies.

As stated by the antecedent meta-analysis, 26 acupoints were withdrawn from the 32 retrieved eligible studies. Therefore, taking Figure 1 as an example, a barplot was presented to sum up the acupoint frequency distribution. The following 10 acupoints were the most frequently selected among all the acupoints for best effects on sleep disorders and similar symptoms: HT7, SP6, PC6, KI1, GV20, EM5, EX-HN3, EX-HN16, KI3, and MA-TF1.

**Figure 2:**
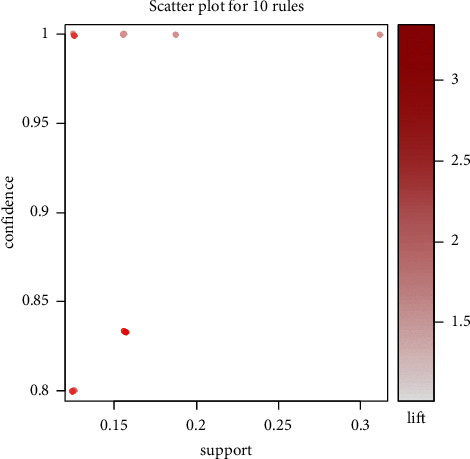
Top 10 association rules presented as a scatter plot.

**Figure 3:**
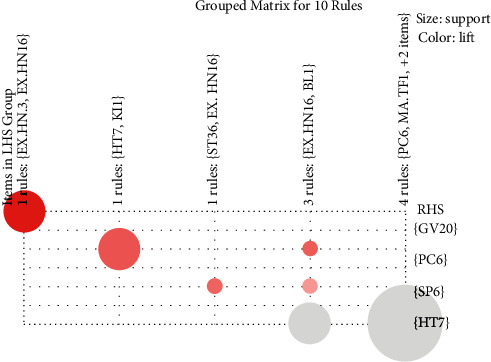
10 association rules presented as grouped matrix.

Data for 26 acupoints were recapitulated from acupoint combinations with reference to association rule analysis for the itemset (Table S1). The sporadic plot in Figure 2 demonstrates that all rules have excellent progress. The support/confidence border can be detected for the optimal rules (i.e., the most interesting rules) [30]. Amidst different acupuncture location points, the association rules are arranged by support. Moreover, the top 10 are listed in Table 1. Color or size is utilized by graph-based visualization to represent the itemset/rules. This plot has two advantages, namely, providing an exceedingly transparent demonstration of rules and enabling very tiny sets of rules to evade cluttered presentation. Subsequently, in Figure 3, the features are visually displayed on the basis of the grouped matrix of 10 associations. As stated in the evidence of the grouped matrix for 10 rules, we can perceive that (EX-HN3, EX-HN16)=>(GV20) and (HT7, KI1)=>(PC6) are interactively selected to uncover the rule's antecedent (LHS) and consequent (RHS) itemset. Through Table 1, we discovered that interactively selected association rules were composed of the No. 6 rule ((HT7, KI1) => (PC6)).

## 4. Discussion

Our results indicate that the core acupoint combinations for treating patients with sleep disorders were (EX-HN3, EX-HN16, GV20) and (HT7, KI1, PC6). With regard to the preceding meta-analysis [26], these acupoint combinations contributed significantly towards the enhancement of PSQI patients under sleep medications with poor sleep quality. Their results demonstrated evidence-based strategies for acupoint selection in the forward therapy. As far as we are aware, this research is the first to point out the potential core acupoint combinations for treating patients with poor sleep quality in accordance with the consequences from a meta-analysis.

The current study corroborates that core acupoint combinations were salutary for patients with sleep disorders. Anti-inflammatory effects [31], improvement in neurologic conditions [32], improvement in hypertension [33], and improvement in exercise tolerance [34] were reported to be viable mechanisms to ameliorate sleep apnea by acupuncture.

Currently, complementary and alternative mediciness are being used worldwide to treat matters that poor sleep quality precipitate as a great public health concern [35–38]. Evaluation of the effects of acupressure on sleep quality can help public health practitioners, clinicians, and patients in making a decision to adopt it as a treatment modality. In developed countries, complementary and alternative medicine treatments are being included in national health insurance packages. On the contrary, acupressure usually remains excluded from these packages. This noninvasive procedure is easy to learn, execute, and is also incredibly convenient. With the help of medical staff, patients and their family members can be taught to implement acupressure on their own. Physicians may suggest acupressure as an incipient treatment to avoid the adverse effects of medications when confronted with recurrent requests from patients for sleeping pill prescriptions during their general practice [39].

Patients were associated with obstructive sleep apnea [40] owing to inflammation, as proved by Boyd et al. Acupuncture treatment may ameliorate both inflammation and sleep quality. A meta-analysis was conducted by Zhao et al. that stated that blood pressure [41] may be attenuated by acupuncture treatment. In addition, Simoncini et al. illustrated that acupuncture treatment with HT7 could boost the neurologic responses [42]. As far as clinical practice is concerned, acupuncture therapy typically provides treatment to patients with acupoint combinations, rather than a single acupoint. There was another suggestion stated by Chen et al. that indicated that acupuncture with multiple acupoints could enhance treatment for cervical spondylosis patients in two ways, namely, better symptomatic improvement and more decline in the regional homogeneity of pain in the matrix area of the brain [43]. Furthermore, as reported by Zhang et al., the acupoint combination of LR3 and KI3 could generate greater synergistic effects in patients with hypertension than that of a single acupoint (LR3 or KI3). The results of resting-state fMRI revealed that acupuncture from LR3 and KI3 could activate broader brain areas in comparison to single LR3 or KI3 [44]. In view of the two advantages of acupoint combination, it is important to ascertain the acupoint combination instead of the single acupoint. This combination could create advancements in the brain area as well as precipitate developments on other related areas of the brain. In addition, the acupoints selected may vary based on the differentiation of symptoms and diseases for the clinician. Meanwhile, TCM treatment is also usually prescribed based on the individual's model diagnosis. A literature review study focused the treatment of insomnia based on TCM pattern differentiation, treatment principle, and pattern-based treatment to investigate the constituency between TCM treatment and acupoints selection [45]. The results showed inconsistence between some differentiations of symptoms in the TCM treatment and acupoints selected. It may be attributed to the insufficient diagnosis process of extracted studies. More high-quality research studies are required to validate that relationship.

To our knowledge, the frequent pattern- (FP-) growth algorithm is the method of finding frequent patterns without candidate generation [46]. The FP-growth algorithm method constructed an FP-tree rather than Apriori using generates and inspects strategy. The FP growth algorithm method concentrated on fracturing the paths of the items and mining frequent patterns. We perform the FP growth algorithm method for sensitivity analysis in this study. We found that results under the FP growth algorithm method (Supplementary Table 3) were similar to Apriori algorithm-based association rule analysis (Table 1). For example, (PC6)=> (HT7) was Top 1 optimal acupuncture association rule determined for both Apriori algorithm and FP growth algorithm-based methods simultaneously. We may conserve the results under the Apriori algorithm method according to sensitivity analysis.

Despite the fact that we explained the core of the study with acupoint combinations, there were a few limitations to our study. First, the effects of acupuncture were affected by several considerations, including the depth of needling, method of acupuncture on manipulation, time of retaining the needle, treatment frequency, and treatment course. However, in this analysis, we did not consider aforementioned factors. Second, this study did not address the roles of other acupuncture systems, such as scalp acupuncture, auricular acupuncture, and Tung's acupuncture. Third, the mechanisms of acupoint combinations remained vague. Owing to these reasons, it is necessary to conduct a thorough evaluation about the further fundamental and clinical studies.

## 5. Conclusions

(EX-HN3, EX-HN16, GV20) integrated with (HT7, KI1, PC6) is deemed as the kernel acupoint combination in the field of acupuncture therapies for sleep disorders. However, due to lack of reproducible verification hitherto, acupuncture is defined as a possible, but not dependable therapy. With reference to our analysis, this kernel acupoint combination was submitted for further verification including clinical trials, basic mechanism research, and treatment strategies.

## Figures and Tables

**Table 1:** Top 10 optimal acupuncture association rules.

No.	Association rules		Support	Confidence	Lift	Expected confidence
1	(PC6)	=> (HT7)	0.31250	1.0000000	1.454545	0.687500
2	(PC6, SP6)	=> (HT7)	0.18750	1.0000000	1.454545	0.687500
3	(EX-HN16)	=> (HT7)	0.15625	1.0000000	1.454545	0.687500
4	(EX-HN3)	=> (GV20)	0.15625	0.8333333	3.333333	0.250000
5	(KI1, PC6)	=> (HT7)	0.15625	1.0000000	1.454545	0.687500
6	(HT7, KI1)	=> (PC6)	0.15625	0.8333333	2.666667	0.312500
7	(ST36)	=> (SP6)	0.12500	1.0000000	2.461538	0.406250
8	(MA-TF1)	=> (HT7)	0.12500	1.0000000	1.454545	0.687500
9	(EX-HN16)	=> (PC6)	0.12500	0.8000000	2.560000	0.312500
10	(EX-HN16)	=> (SP6)	0.12500	0.8000000	1.969231	0.406250

## Data Availability

The data used to support the findings of this study are included within the article.

## Supplementary Materials

Supplementary Materials Table 1: summary of 26 acupuncture point locations involved in studies as binary data. Table 2: quality assessment with overall bias. Figure 1: summary of risk of bias plot of 13 RCTs.
