# Supplementary material for: An Association Rule Analysis of the Acupressure Effect on Sleep Quality
Source: Evid Based Complement Alternat Med. 2021 Sep 29;2021:1399258. doi: 10.1155/2021/1399258 (PMC8494578; doi:10.1155/2021/1399258)
Supplement: Supplementary Materials — Table 1: summary of 26 acupuncture point locations involved in studies as binary data. Table 2: quality assessment with overall bias. Figure 1: summary of risk of bias plot of 13 RCTs. [file 1399258.f1.zip › 1399258.f1/SUPPLEMENTARY DESCRIPTION_1399258.docx]

**SUPPLEMENTARY DESCRIPTION:**

**Supplementary Table 1:** Summary of 26 acupuncture point locations involved in studies as binary data.

**Supplementary Table 2:** Quality assessment with overall bias.

**Supplementary Table 3**. Top 10 FP-Growth algorithm-based association rules of acupunctures

**Supplementary Figure 1:** Summarize risk of bias plot of 13 RCTs.
