# Supplementary material for: An Association Rule Analysis of the Acupressure Effect on Sleep Quality
Source: Evid Based Complement Alternat Med. 2021 Sep 29;2021:1399258. doi: 10.1155/2021/1399258 (PMC8494578; doi:10.1155/2021/1399258)
Supplement: Supplementary Materials — Table 1: summary of 26 acupuncture point locations involved in studies as binary data. Table 2: quality assessment with overall bias. Figure 1: summary of risk of bias plot of 13 RCTs. [file 1399258.f1.zip › 1399258.f1/Supplementary_Tables_(ECAM)_edit_20210912.docx]

**Supplementary Table 1.** 26 acupuncture point locations involved in studies

| Studies/Locations | EX-HN16 | BL1 | BL2 | BL10 | BL18 | BL23 | MA-TF1 | GB20 | GB21 | GB34 | GV20 | GV23 | GV24 | HT7 | KI1 | KI3 | LI4 | LR3 | PC6 | SP6 | EX-HN1 | ST8 | ST36 | EM5 | TE23 | EX-HN 3 |
| --- | --- | --- | --- | --- | --- | --- | --- | --- | --- | --- | --- | --- | --- | --- | --- | --- | --- | --- | --- | --- | --- | --- | --- | --- | --- | --- |
| Abedian, 2015 | 0 | 0 | 0 | 0 | 0 | 0 | 0 | 1 | 0 | 0 | 0 | 0 | 0 | 1 | 0 | 0 | 0 | 0 | 0 | 1 | 0 | 0 | 0 | 0 | 0 | 1 |
| Arab, 2015 | 0 | 0 | 0 | 0 | 0 | 0 | 0 | 0 | 0 | 0 | 0 | 0 | 0 | 1 | 0 | 0 | 0 | 0 | 0 | 0 | 0 | 0 | 0 | 0 | 0 | 0 |
| Chen ML, 1999 | 1 | 0 | 0 | 0 | 0 | 0 | 1 | 0 | 0 | 0 | 1 | 0 | 0 | 1 | 0 | 0 | 0 | 0 | 0 | 0 | 0 | 0 | 0 | 0 | 0 | 0 |
| Lei, 2015 | 1 | 0 | 0 | 0 | 0 | 0 | 0 | 0 | 0 | 0 | 1 | 0 | 0 | 1 | 0 | 0 | 0 | 0 | 1 | 1 | 0 | 0 | 0 | 1 | 0 | 0 |
| Liu, 2012 | 0 | 0 | 0 | 0 | 0 | 0 | 0 | 0 | 0 | 0 | 0 | 0 | 0 | 1 | 1 | 0 | 0 | 0 | 1 | 0 | 0 | 0 | 0 | 1 | 0 | 0 |
| Lu, 2013 | 0 | 0 | 0 | 0 | 0 | 0 | 0 | 0 | 0 | 0 | 0 | 0 | 0 | 1 | 1 | 0 | 0 | 0 | 1 | 0 | 0 | 0 | 0 | 0 | 0 | 0 |
| Nordio, 2008 | 0 | 0 | 0 | 0 | 0 | 0 | 0 | 0 | 0 | 0 | 0 | 0 | 0 | 1 | 0 | 0 | 0 | 0 | 0 | 0 | 0 | 0 | 0 | 0 | 0 | 0 |
| Reza 2010 | 1 | 0 | 0 | 0 | 0 | 0 | 1 | 0 | 0 | 0 | 0 | 0 | 0 | 1 | 1 | 0 | 0 | 0 | 1 | 1 | 0 | 0 | 0 | 0 | 0 | 0 |
| Shariati, 2012 | 0 | 0 | 0 | 0 | 0 | 0 | 0 | 0 | 0 | 0 | 0 | 0 | 0 | 1 | 0 | 0 | 1 | 0 | 0 | 1 | 0 | 0 | 0 | 0 | 0 | 0 |
| Tang, 2014 | 0 | 0 | 0 | 0 | 0 | 0 | 0 | 0 | 0 | 0 | 0 | 0 | 0 | 0 | 0 | 0 | 1 | 0 | 0 | 1 | 0 | 0 | 1 | 0 | 0 | 0 |
| Tsay, 2003 | 0 | 0 | 0 | 0 | 0 | 0 | 1 | 0 | 0 | 0 | 0 | 0 | 0 | 1 | 1 | 0 | 0 | 0 | 0 | 0 | 0 | 0 | 0 | 0 | 0 | 0 |
| Zheng, 2014 | 0 | 0 | 0 | 0 | 0 | 0 | 0 | 0 | 0 | 0 | 0 | 0 | 0 | 1 | 0 | 1 | 0 | 0 | 0 | 0 | 0 | 0 | 0 | 0 | 0 | 0 |
| Zhou 2010 | 0 | 1 | 0 | 0 | 0 | 0 | 0 | 0 | 0 | 0 | 1 | 0 | 0 | 0 | 0 | 0 | 0 | 0 | 0 | 0 | 0 | 0 | 0 | 1 | 0 | 1 |
| Buguet, 1995 | 0 | 0 | 0 | 0 | 0 | 0 | 0 | 0 | 0 | 0 | 0 | 0 | 0 | 1 | 0 | 0 | 0 | 0 | 0 | 0 | 0 | 0 | 0 | 0 | 0 | 0 |
| Cai, 2012 | 0 | 0 | 0 | 0 | 0 | 0 | 0 | 0 | 0 | 0 | 0 | 0 | 0 | 0 | 1 | 1 | 0 | 0 | 0 | 0 | 0 | 0 | 0 | 0 | 0 | 0 |
| Chen, 2013 | 0 | 0 | 0 | 0 | 1 | 0 | 0 | 0 | 0 | 0 | 0 | 0 | 0 | 1 | 0 | 0 | 0 | 0 | 0 | 1 | 0 | 0 | 0 | 0 | 0 | 0 |
| Dai, 2007 | 0 | 0 | 0 | 0 | 0 | 0 | 0 | 0 | 0 | 0 | 0 | 0 | 0 | 0 | 1 | 1 | 0 | 0 | 0 | 1 | 0 | 0 | 1 | 0 | 0 | 0 |
| He, 2011 | 0 | 0 | 1 | 0 | 0 | 0 | 0 | 1 | 1 | 0 | 1 | 1 | 0 | 0 | 0 | 0 | 0 | 0 | 0 | 0 | 0 | 1 | 0 | 1 | 1 | 1 |
| Huang, 2014 | 0 | 0 | 0 | 0 | 0 | 0 | 0 | 0 | 0 | 0 | 0 | 0 | 0 | 1 | 0 | 0 | 0 | 0 | 1 | 1 | 1 | 0 | 1 | 0 | 0 | 0 |
| Jin, 2015 | 0 | 0 | 0 | 0 | 0 | 0 | 0 | 0 | 0 | 0 | 0 | 0 | 0 | 0 | 1 | 0 | 0 | 0 | 0 | 1 | 0 | 0 | 0 | 0 | 0 | 0 |
| Li, 2014 | 1 | 0 | 0 | 0 | 0 | 0 | 0 | 0 | 0 | 0 | 0 | 0 | 0 | 1 | 0 | 0 | 0 | 0 | 1 | 1 | 0 | 0 | 0 | 1 | 0 | 0 |
| Liao, 2013 | 0 | 0 | 1 | 0 | 0 | 0 | 0 | 0 | 1 | 0 | 1 | 0 | 1 | 0 | 0 | 0 | 0 | 0 | 0 | 0 | 0 | 0 | 0 | 0 | 0 | 1 |
| Liu, 2010 | 0 | 0 | 0 | 0 | 0 | 1 | 0 | 0 | 0 | 0 | 1 | 0 | 0 | 1 | 0 | 1 | 0 | 0 | 1 | 1 | 0 | 0 | 0 | 0 | 0 | 0 |
| Liu, 2014 | 0 | 0 | 0 | 0 | 0 | 0 | 0 | 0 | 0 | 0 | 1 | 0 | 0 | 0 | 0 | 0 | 0 | 0 | 0 | 0 | 0 | 0 | 0 | 0 | 1 | 1 |
| Mei, 2014 | 0 | 0 | 0 | 0 | 0 | 0 | 0 | 1 | 0 | 1 | 0 | 0 | 0 | 0 | 0 | 0 | 0 | 0 | 0 | 1 | 0 | 0 | 1 | 0 | 0 | 0 |
| Nasiri, 2011 | 0 | 0 | 0 | 0 | 0 | 0 | 1 | 0 | 0 | 0 | 0 | 0 | 0 | 1 | 1 | 0 | 0 | 0 | 1 | 0 | 0 | 0 | 0 | 0 | 0 | 0 |
| Qiu, 2000 | 0 | 0 | 1 | 0 | 0 | 0 | 0 | 1 | 0 | 0 | 1 | 1 | 0 | 0 | 0 | 0 | 0 | 0 | 0 | 0 | 0 | 0 | 0 | 1 | 1 | 1 |
| Sun, 2010 | 0 | 0 | 0 | 0 | 0 | 0 | 0 | 0 | 0 | 0 | 0 | 0 | 0 | 1 | 0 | 0 | 0 | 0 | 0 | 0 | 0 | 0 | 0 | 0 | 0 | 0 |
| Wu, 2015 | 0 | 0 | 0 | 0 | 0 | 0 | 0 | 0 | 0 | 0 | 0 | 0 | 0 | 1 | 0 | 1 | 0 | 1 | 0 | 0 | 0 | 0 | 0 | 0 | 0 | 0 |
| Yang, 2015 | 0 | 0 | 0 | 0 | 0 | 0 | 0 | 0 | 0 | 0 | 0 | 0 | 0 | 1 | 0 | 0 | 0 | 0 | 0 | 0 | 0 | 0 | 0 | 0 | 0 | 0 |
| Zeng, 2012 | 1 | 0 | 0 | 0 | 0 | 0 | 0 | 0 | 0 | 0 | 0 | 0 | 0 | 1 | 0 | 0 | 0 | 0 | 1 | 1 | 0 | 0 | 0 | 1 | 0 | 0 |
| Zeng, 2014 | 0 | 0 | 0 | 1 | 0 | 0 | 0 | 0 | 0 | 0 | 0 | 0 | 0 | 1 | 1 | 0 | 0 | 0 | 1 | 0 | 0 | 0 | 0 | 0 | 0 | 0 |

**Supplementary Table 2.** Quality assessment with overall bias

| Study | Random sequence generation | Allocation concealment | Blinding of participants and personnel | Blinding of outcome assessment | Incomplete outcome data | Selective reporting | Compliance bias | Overall |
| --- | --- | --- | --- | --- | --- | --- | --- | --- |
| Abedian, 2015 | Low | Low | Low | Low | Low | Low | Unclear | Low |
| Arab, 2015 | Low | Low | Unclear | Low | Low | Low | Unclear | Low |
| Chen ML, 1999 | Low | Unclear | High | Low | Low | Low | Unclear | Low |
| Lei, 2015 | Low | Unclear | High | Low | Low | Low | Unclear | Low |
| Liu, 2012 | Low | Unclear | High | Unclear | Low | Low | Unclear | Low |
| Lu, 2013 | Low | Unclear | High | Unclear | Low | Low | Unclear | Low |
| Nordio, 2008 | Low | Unclear | High | Unclear | Low | Low | Unclear | Low |
| Reza 2010 | Low | Unclear | High | High | Low | Low | Unclear | Low |
| Shariati, 2012 | Low | Unclear | High | High | Low | Low | High | Low |
| Tang, 2014 | Unclear | Unclear | High | High | Low | Low | High | Low |
| Tsay, 2003 | Unclear | Unclear | High | High | High | Low | High | High |
| Zheng, 2014 | Unclear | Unclear | High | High | High | Low | High | High |
| Zhou 2010 | Unclear | High | High | High | High | Unclear | High | High |

**Supplementary Table 3**. Top 10 FP-Growth algorithm-based association rules of acupunctures

| No | Association rules | Confidence | Lift | Leverage | Conviction |
| --- | --- | --- | --- | --- | --- |
| 1 | [PC6=1] => [HT7=1] | 1.00 | 1.45 | 0.10 | 3.13 |
| 2 | [EX-HN16=1] => [HT7=1] | 1.00 | 1.45 | 0.05 | 1.56 |
| 3 | [MA-TF1=1] => [HT7=1] | 1.00 | 1.45 | 0.04 | 1.25 |
| 4 | [ST36=1] => [SP6=1] | 1.00 | 2.46 | 0.07 | 2.38 |
| 5 | [SP6=1, PC6=1] => [HT7=1] | 1.00 | 1.45 | 0.06 | 1.88 |
| 6 | [SP6=1, EX-HN16=1] => [HT7=1] | 1.00 | 1.45 | 0.04 | 1.25 |
| 7 | [PC6=1, KI1=1] => [HT7=1] | 1.00 | 1.45 | 0.05 | 1.56 |
| 8 | [HT7=1, EM5=1] => [PC6=1] | 1.00 | 3.20 | 0.09 | 2.75 |
| 9 | [PC6=1, EM5=1] => [HT7=1] | 1.00 | 1.45 | 0.04 | 1.25 |
| 10 | [PC6=1, EX-HN16=1] => [HT7=1] | 1.00 | 1.45 | 0.04 | 1.25 |
